# Systematic review on the rational use of amniotic membrane allografts in diabetic foot ulcer treatment

**DOI:** 10.1186/s12893-021-01084-8

**Published:** 2021-02-15

**Authors:** Kasun Lakmal, Oshan Basnayake, D. Hettiarachchi

**Affiliations:** Department of Anatomy, Faculty of Medicine, University of Colombo, 25, Kynsey Place 8, Colombo, Sri Lanka

**Keywords:** Amniotic membrane, Diabetes, Foot ulcers, Allografts

## Abstract

**Background:**

Diabetic foot ulcer is a complication with multiple aetiological factors which has a significant impact to patients’ lives and costs to the healthcare system. The potential of human amniotic membrane to act as an allograft has been studied in relation to this condition. Aim of this study is to evaluate the current scientific evidence on its effectiveness in healing diabetic foot ulcers.

**Methods:**

Pubmed, Cochrane library, and Google scholar were searched using the search terms, “Amnion” OR “Placenta” AND “Diabetic foot”. (MeSH terms) in the title or the abstract field from 1st of January 2000 to 30th March 2020. The quality of published reports was assessed using standard methods. We searched for experimental and observational studies in terms of randomized control trials, prospective cohort, retrospective cohort studies and case series.

**Results:**

When searched with Mesh terms, 12 citations in PubMed, 22 citations in Cochrane library and 30 in other data bases were found. After screening the studies and their reference lists, 12 studies met the inclusion criteria and the others were excluded. There were 8 randomized control trials (RCTs), 2 prospective studies and 2 retrospective studies employing different preparation methods of the amniotic membranes. A wide variation in study end points were noted. Majority of the RCTs (n = 7) were concluded with significantly higher wound closure rate compared to the conventional treatment groups. In prospective and retrospective studies, it was shown that large chronic ulcers which were resistant to closure with standard therapy achieved wound closure with amniotic membrane allografts. A meta-analysis could not be performed due to study heterogeneity, and publication bias was not assessed due to the small number of available studies which was not sufficient for accurate comparison.

**Conclusion:**

Even though, the studies had some inherent heterogeneity due to different preparation methods, different study end points and outcome measurements. According to our review the current studies using amniotic membrane allografts give reliable evidence of reduction in healing time over conventional methods.

## Background

The human amniotic membrane has shown immense potential as an allograft. Owing to its several unique qualities such as a rich milieu of amino acids, growth factors and other nutrients that facilitates its intrauterine function as it forms the feto-maternal interphase. Human Amniotic Allograft Membrane (HAA) can support wound healing by facilitating cell migration and promoting repair [1]. One such use is in the treatment of chronic wounds, in the early twentieth century this possibility was explored and further expanded to diabetic neurovascular ulcers. The recent development of gamma irradiated or dehydrated amniotic membrane grafts has enabled us to bypass some of the drawbacks experienced with traditional graphing method including issues with storage and preparation [2].

Diabetic foot ulcers (DFU) are estimated to affect 15% of diabetic patients. They experience foot ulcers once in their lifetime with a recurrence rate of 35–50% over 3 years to around 70% over 5 years [2–4]. Complications of diabetic foot ulcers maybe related to its chronicity, osteomyelitis, re-ulceration, gangrene and amputation which might be aggravated by concomitant co-morbidities such as peripheral vascular disease, sub-optimal blood glucose control and neuropathy to name a few [5]. The long-drawn healing process in a DFU make them more susceptible for infection and resulting complications leading to healthcare economic burden [6]. The standard care for a DFU includes management of infections, local wound care offloading (especially in DFU complicated with neuropathy) and correcting systemic factors to promote healing. Some clinicians recommend advanced treatment such as biological dressings, collagen, platelet-derived growth factors (PDGF), and platelet-rich plasma (PRP) for non-healing ulcers after a\standard wound care [1]. In this light natural amniotic membrane wound dressings have been used for over a century as it contains a single epithelial cell layer, a thick basement membrane and an avascular stroma making it an ideal biological graft. Human amniotic membrane can assist in wound healing by cell migration into the healing tissue. Acquiring placenta for the harvesting of amniotic membrane is a challenge in terms of ethical aspects and the harvesting, processing, and preservation of the membrane as biological dressing are expensive procedures. Products containing amniotic tissue are increasingly being manufactured either as cryopreserved or dehydrated grafts [7]. We sought to investigate the rational use of amniotic membrane allografts in the management of diabetic foot ulcers by conducting a systemic review through published studies. Objective of the study was to assess the impact on wound closure rates by the use of amniotic membrane in diabetic foot ulcers.

## Methods

PubMed, Cochrane library, CINAHL, Embase, Web of Science, and Clinicaltrials.gov and Google scholar engines were searched for the terms “Amnion” OR “Placenta” AND “Diabetic foot” (MeSH terms) in the title or in the abstract field from 1st of January 2000 to 30^th^ March 2020. A non-English language database known as APAMED central was searched using the same criteria to reduce the publication bias. The reference lists provided in full papers were also used to identify additional papers for review. Quality of published reports was assessed using Downs and Black checklist. Downs and Black score ranges were given corresponding quality levels as previously reported [8]: excellent (26–28); good (20–25); fair (15–19); and poor (≤ 14). Additionally authors attempted to reduce the publication bias and between-study heterogeneity by employing standard methods such as extended funnel plot tests for detecting publication bias, and selection modelling and trim-and-fill methods to adjust for publication bias in the presence of between-study heterogeneity.

**Fig. 1:**
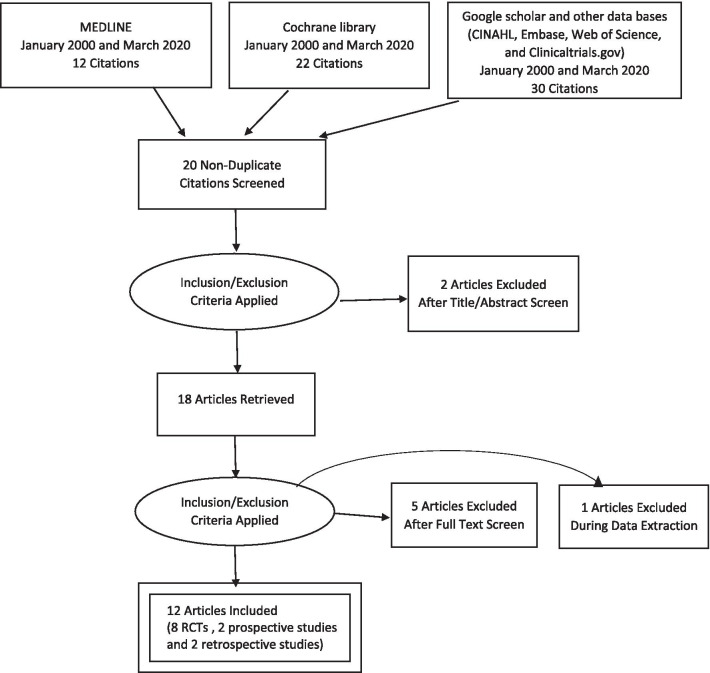
Prisma flow chart

We searched for experimental and observational studies in terms of randomized control trials, prospective cohorts, and retrospective cohort studies. Case reports were excluded from this review. Only studies pertaining to human subjects were selected. The primary objective of this systematic review was to identify the outcomes of the use of amniotic membrane in the rate of healing in diabetic foot ulcers (Fig. 1).

Initial eligibility screening was performed based on the titles and abstract from electronic databases. Thereafter, the full text papers of all studies were assessed based on the inclusion and exclusion criteria. In doubtful situations the opinion of the senior investigator was sought. The studies done with both type 1 and type 2 diabetes patients were included. The studies which have used different preparation of amniotic allografts (dehydrated, cryopreserved and stem cell extractions) were included. When including the RCTs, studies which compared the amniotic membrane treatment with standard or conventional care were selected. Studies that are designed with the aim of analyzing the molecular basis without measuring clinical improvement of the ulcers were also excluded from our study. From each study data were extracted on trial design, study setting, amniotic membrane preparation methods used, control interventions, outcome measures and statistical analysis. Outcome measures were extracted in terms of the healing time, healed percentage, recurrences and adverse outcomes.

## Results

When searched with Mesh terms 12 citations in Pubmed, 22 citations in Cochrane library and 30 in other data bases were found. We couldn’t find new studies by going through reference lists. By screening the studies total of 12 non-duplicated studies met the inclusion and exclusion criteria. There were 8 randomized control trials, 2 prospective studies and 2 retrospective studies (Fig. 1). Even though the search was done from the studies conducted since 2000, all the studies that met the criteria and included in the review were done in the last decade i.e. after 2010. We found 8 randomized control trials [1, 2, 5, 9–13] and all were performed in the United States and five of those were multicenter trials. Out of the 2 prospective studies one was done in Spain [14] and the other in the United States [15]. Both retrospective studies [16, 17] were also performed in the United States. According to the Downs and Black scoring system, 4 studies [5, 9, 11, 13] were graded as “Good” (score ranging from 20 to 25) and rest of the 8 studies were graded as ‘Fair” (15–19).

There were total 244 participants in intervention groups and 210 in the control groups of 8 randomized control trials, except in in one study. Total of 28 in prospective and total of 92 in retrospective studies were treated with amniotic membrane preparations. The mean duration of the diabetes mellitus in the participants was reported only in one study [13]. There were patients with different ulcer locations in their feet and in all the above studies ulcer duration was more than 28 days. Mean size of the ulcers in prospective and retrospective studies were more than 5 cm^2^ and it was less than 5 cm^2^ in majority of participants in randomized control trials (Table 1). Different amniotic membrane preparations have been used (Amnioband [9], AMNIOEXCEL [10, 15], Epifix [2,11,12,], Apligraf [11], Grafix [13], NEOX CORD [16], (dHACM) [5, 17]).

**Table 1:** Characteristics of study groups

	Author	Year	Location (setting)	Study type	Study size	Mean age (years)/SD	Mean duration of DM	Ulcer location/s	Mean ulcer duration (days)/SD	Mean ulcer size (cm^2^)/SD
1	Thompson et al. [1]	2019	North Dakota USA	RCT	13 (I = 7, C = 6)	I = 58.5(12.96) C = 55.17(18.32	NA	Plantar	NA	I = 1.54(1.74) C = 2.78(3.04)
2	Tettebach et al. [5]	2019	Multicenter USA	RCT	110 (I = 54,C = 56)	I = 57.4(10.6) C = 57.1(10.5)	NA	Toe, forefoot, midfoot and hindfoot	I = 145.6(129.5) C = 149.8(110.6)	I = 3.2(2.8) C = 3.9(3.8)
3	Didomenico et al. [9]	2016	Multicenter USA	RCT	40 (I = 20, C = 20)	I-59.0(13) C-58.0(9)	NA	Toe, forefoot, midfoot, heel, ankle, and hindfoot	> = 28.0	I = 2.0(0.90) C = 3.3(4.35)
4	Snyder et al. [10]	2016	Multicenter USA	RCT	29 (I = 15, C = 14)	I = 57.9(12.49) C = 58.6(6.97)	NA	Forefoot, midfoot, hindfoot, pahanges, metatarsals	> = 28.0	I = 4.7(5.43) C = 6.9(6.75)
5	Zelen et al [11]	2015	Multicenter USA	RCT	100 (I_1_ = 32, I_2_ = 33, C = 35)	I_1_ = 63.3(12.25) I_2_ = 63.8(11.86) C = 60.6(11.55)	NA	Toe, forefoot, midfoot hindfoot, and ankle	I_1_ = 121.1(107.1) I_2_ = 133.0(103.46) C = 98.7(90.3)	I_1_ = 2.6(2.97) I_2_ = 2.7(2.75) C = 3.1(3.17)
6	Zelen et al. [12]	2014	Southwest Virginia USA	RCT	40 (I = 20, C = 20)	I = 59.6(13.8) C = 60.8(0.9)	NA	Toe, forefoot, midfoot and hindfoot	I = 118.3(151.9) C = 122.5(101.5)	I = 2.4(1.8) C = 2.0(1.3)
7	Lavery et al. [13]	2014	Multicenter USA	RCT	97 (I = 50, C = 47)	I = 55.5(11.5) C = 55.1(12.0)	I = 15.4(11.1) C = 14.0(11.0)	Dorsal and plantar	I = 115.0(72.6) C = 122.9(83.9)	I = 3.41(3.23) C = 3.93(3.22)
8	Zelen et al. [2]	2013	Southwest Virginia USA	RCT	25 (I = 13, C = 12)	I = 56.4(14.7) C = 61.7(10.3)	NA	Forefoot, digital, heel and midfoot	I = 98.7(91.0) C = 114.8(108.5)	I = 2–6(1.9) C = 3.4(2.9)
9	Valiente et al. [14]	2018	El Palmar Spain	Prospective Case series	N = 14	57	NA	Forefoot, midfoot and hind foot	> = 56	12.30
10	Abdo [15]	2016	St.Louis USA	Prospective Cases series	N = 14	56.7(9.1)	NA	Forefoot, midfoot and hindfoot	> = 28	6.5(11.6)
11	Raphael[16]	2016	Georgia USA	Retrospective study	N = 29	52.9(1.83)	NA	Forefoot., midfoot and hindfoot	340.2(116.9)	10.6(2.15)
12	Kirsner et al. [17]	2015	Multicenter USA	Retrospective study	N = 63	61.1(12.2)	NA	Planter	128.8	5.2

*SD* standard deviation, *DM* diabetes mellitus, *RCT* randomized control trial, *I* intervention group, *C* control group, *NA* not available

In 6 randomized control trials the follow up duration was 12 weeks and in the rest, it was 6 weeks. Both prospective and one retrospective study [16] included data until complete wound closure was achieved. Majority of randomized control trials (n = 7) have demonstrated statistically significant closure rates at the study endpoint compared to conventional or standard wound care procedures (p < 0.05). Adverse graft outcomes were low in studies where safety evaluation data was available (n = 6). One study [13] showed statistically significant low infection rate in the intervention group (p < 0.044). Thompson et al. evaluated 90-day recurrence rates in both intervention and control group and a lower recurrence rate was observed in the intervention group (14.29% versus 83.3%) similarly Tettlebatch et al. also showed a lower recurrence rate at 112 days (5% versus 14%).

In one prospective study [14], the mean duration of ulcer was more than 56 days and the mean ulcer size was 12.30 cm^2^ in comparison the other prospective study [15] these two parameters were more than 28 days and 6.5 cm^2^. Median ulcer closure times were 20 weeks and 5 weeks, respectively. The mean ulcer duration was longest in one retrospective study [16], which was 340 days with mean ulcer size of 10.6 cm^2^. This study concluded that the median ulcer closure time was 9 weeks. In the other retrospective study [17], mean ulcer duration was 128.8 days and mean ulcer size was 5.2cm^2^ and this study demonstrated that the median time of healing was 26 weeks (Table 2).

**Table 2:** Interventions and outcomes of the studies

	Author	Year	Study type	Intervention and size of the group	Evaluation frequency	Follow-up time	Healing time(days) /(SD)	Healed percentage	Recurrences	Adverse outcomes. (amniotic membrane product related)
1	Thompson et al. [1]	2019	RCT	Human amniotic allograft 7 SOC-6	Weekly	12 weeks	I-29.50(15.41) C- 26.20(8.93) (p value has not been calculated due to small sample size)	NA	90-day recurrence rate I-14.29% C-83.3%	NA
2	Tettebach et al. [5]	2019	RCT	dHACM – 54 SOC- 56	Weekly	12 weeks	P = 0.0187 (Kaplan–Meier plot of time to heal)	I-70% C-50% P = 0.0338	112-day recurrence I-5% C-14%	3 product related events
3	Didomenico et al. [9]	2016	RCT	AmnioBand 20 SOC 20	Weekly	12 weeks	I – 36.0 C – 70.0 P = 0.00073	I-85%, C-25% P = 0.00073 Odds ratio = 17	NA	NA
4	Snyder et al. [10]	2016	RCT	AMNIOEXCEL 15 SOC 14	Weekly	6 weeks	NA	I-35%, C-0% P = 0.017	NA	Not observed
5	Zelen et al.[11]	2015	RCT	EpiFIx 32 Apligraf 33 SOC 35	Weekly	12 weeks	I_1_-23.6 I_2_- 47.9 C = 57.4	I_1_-97% I_2_-73% C-51 P = 0.0019	NA	Not observed
6	Zelen et al. [12]	2014	RCT	Epifix 20 SOC 20	I – weekly C—biweekly	12 weeks	I-16.8(12.6) C-29.0(17) P = 0.039	I-85% C-100%	NA	Not observed
7	Lavery et al. [13]	2014	RCT	Grafix 50 SCO 47	Weekly	12 weeks	I-42.0 C-69.5 P = 0.019	I-62.0% C-21.3% P = 0.0001	NA	Would infections were low in interventional group (p = 0.044)
8	Zelen et al. [2]	2013	RCT	Epifix 13 SOC 12	Weekly	6 weeks	I-17.5(13.3) C-35.0	I-92% C-8% P = 0.0001	NA	NA
9	Valiente et al. [14]	2018	Prospective Case series	Cryopreserved amniotic membrane 14	Weekly	Until complete closure	Median time 20 weeks (range 7–56)	NA	NA	Not observed
10	Abdo [15]	2016	Prospective Cases series	AMNIOEXCEL 14	Weekly	Until complete closure	Median 5 weeks (range 1014 weeks)	NA	NA	NA
11	Raphael [16]	2016	Retrospective study	NEOX CORD 1 K (cryopreserved amniotic membrane) 29	Weekly	Until complete closure	Mean 96.6(13.65) days Median 9 weeks	87.5%	NA	NA
12	Kirsner et al. [17]	2015	Retrospective study	dHACM 63	NA	24 weeks	Median time 26 weeks	At 12 weeks-28% At 24 weeks-47%	NA	NA

*SD* standard deviation, *RCT* randomized control trials, *SOC* standard of care, *I* intervention group, *C* control group, *dHACM* dehydrated human amnion/chorionic membrane

## Discussion

This study aimed to evaluate the current scientific evidence on effectiveness of use of amniotic membrane in healing the diabetic foot ulcers. In the analysis of retrospective studies, majority of the RCTs (n = 7) were concluded with significantly higher wound closure rates compared to the conventional treatment group. One randomized control trial showed less recurrent rate of the healed ulcers after treatment. In prospective and retrospective studies showed that larger and more chronic ulcers which are resistant to close with the standard therapy achieve wound closure with amniotic membrane allografts. Minimal numbers of adverse effects attributable to amniotic membrane product were observed in the included studies.

Only two RCTs aimed at assessing the recurrence rate following total closure of the ulcers [1, 5]. Follow up details were not included in the other studies in terms of recurrent rates and further complications. Amniotic membrane preparations used in different studies were different to each other. Currently commercially available amniotic membranes are expensive and median graft costs in some studies were between 2000 and 10,000 of dollars [11, 12]. The main limitations of these studies were the heterogeneity study methods and outcomes, limited number of RCTs and small number of the of study participants. Except one study [14] other studies were conducted in the USA limiting the generalization of the results to the global population. A meta-analysis could not be performed due to study heterogeneity, and publication bias was not assessed due to the small number of available studies for each comparison. Furthermore, this study findings are in-line with previously conducted study on the efficacy and time sensitivity of human amnion/chorion membrane treatment in patients with diabetic foot ulcers which concluded that when amniotic membranes were combined with standard care diabetic foot ulcers healed significantly faster than standard care alone [18]. However, we recommend that further prospective randomized control trials with larger population with long term follow-up have to be performed for better evidence. The current evidence suggests the use of amniotic membrane preparations for resistant diabetic foot ulcers can achieve relatively fast wound closure rates.

## Conclusions

According to our review the current studies summarize reliable evidence to suggest reduction in healing time with amniotic membrane preparations in the treatment of refractory chronic diabetes foot ulcers compared to conventional methods.

## Data Availability

Not applicable.

## Abbreviations

DFU: Diabetic foot ulcer
dHACM: Dehydrated human amniotic chorionic membrane
DM: Diabetes mellitus
HAA: Human amniotic allograft membrane
NA: Not available
PDGF: Platelet Derived Growth Factors
PRP: Platelet-rich Plasma
RCT: Randomized control trials
SD: Standard deviation
SOC: Standard of care
USA: Untied States of America
